# Supplementary material for: Pten regulates collagen fibrillogenesis by fibroblasts through SPARC
Source: PLoS One. 2021 Feb 3;16(2):e0245653. doi: 10.1371/journal.pone.0245653 (PMC7857610; doi:10.1371/journal.pone.0245653)
Supplement: S1 Fig — (PDF) [file pone.0245653.s001.pdf]

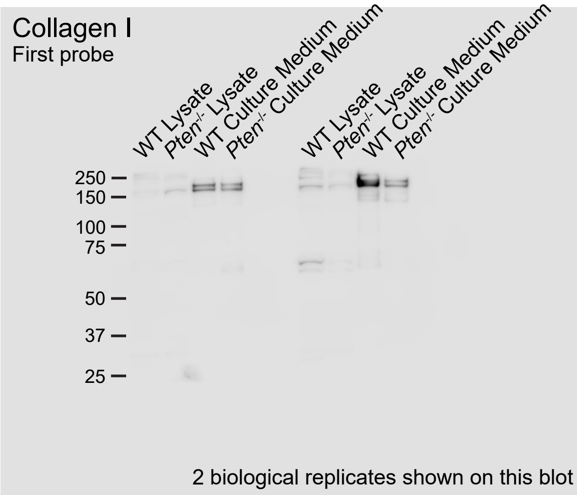

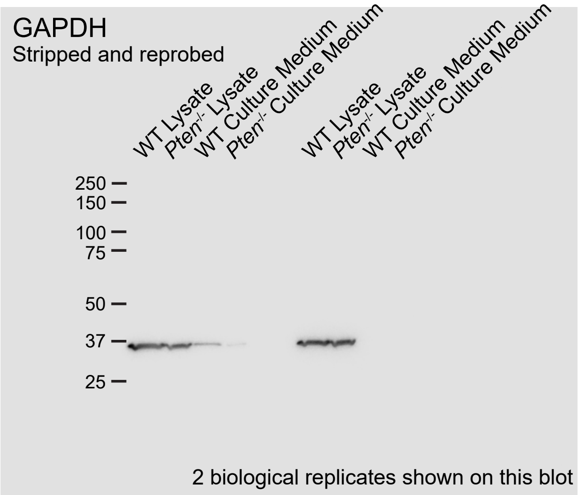

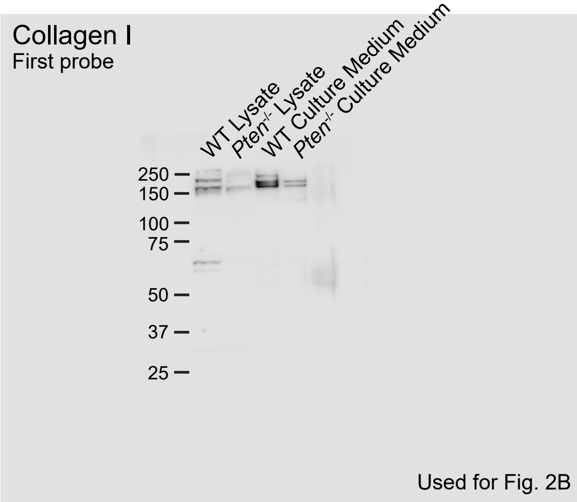

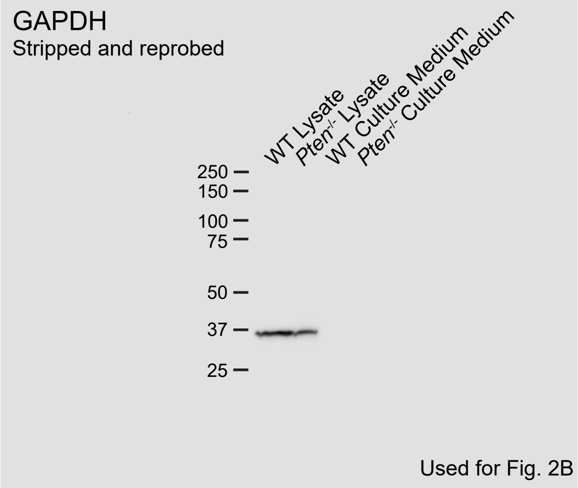

Images captured using LI-COR Odyssey FC imaging system.

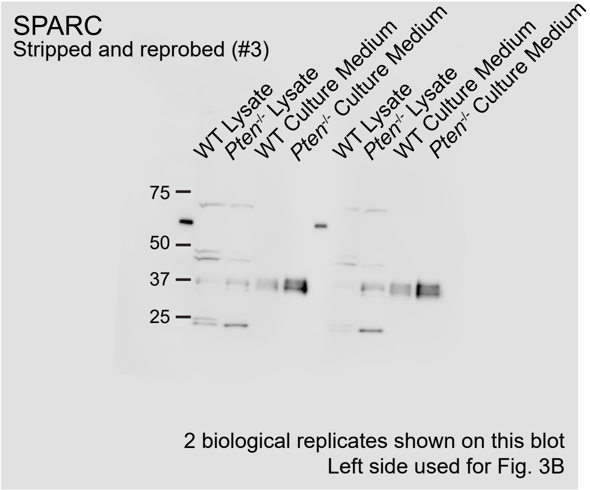

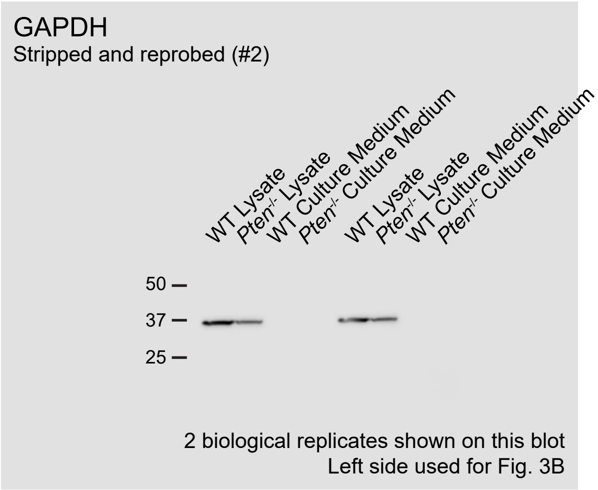

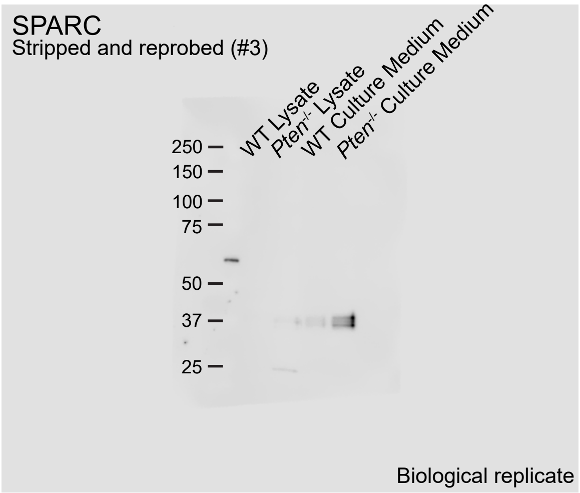

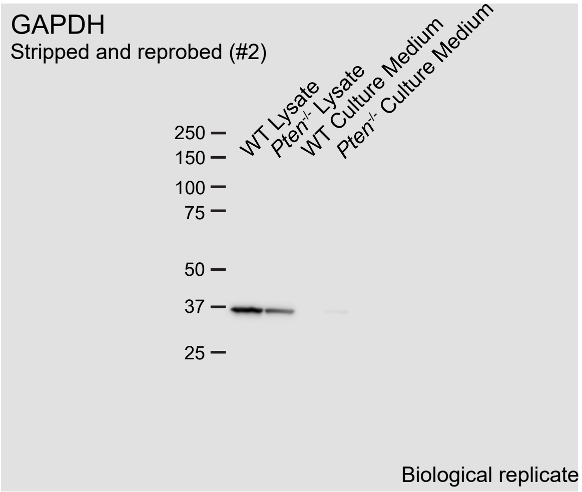

Images captured using LI-COR Odyssey FC imaging system.

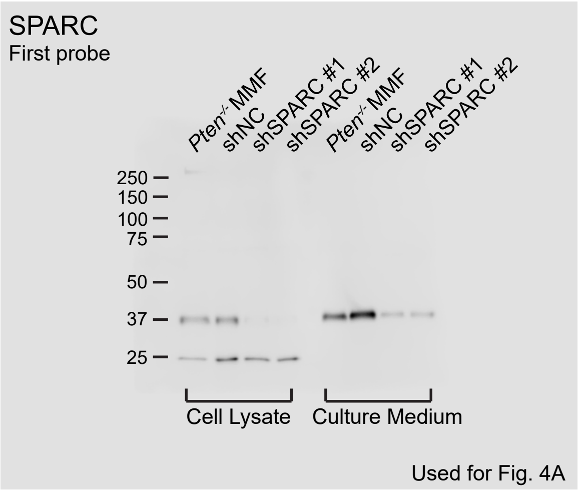

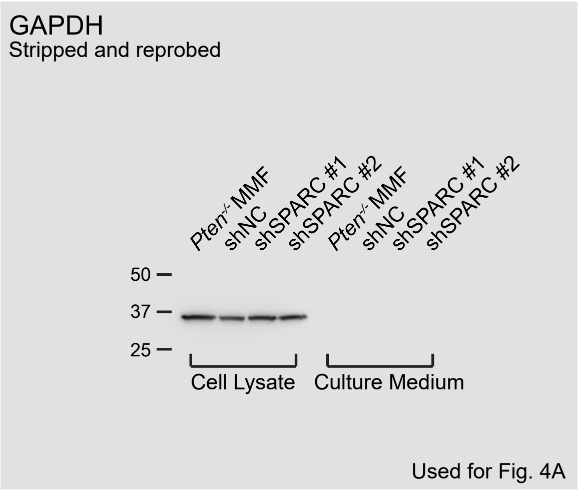

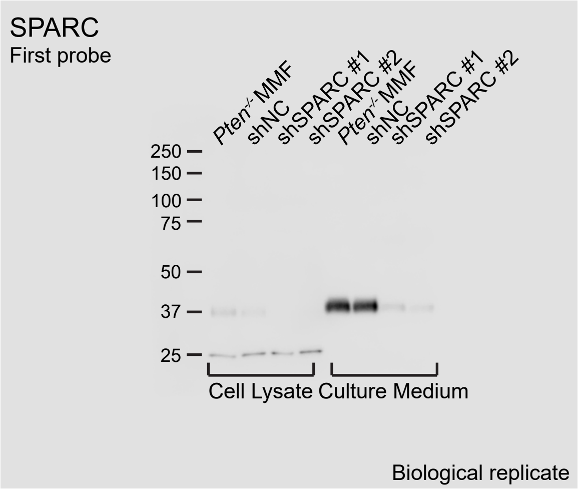

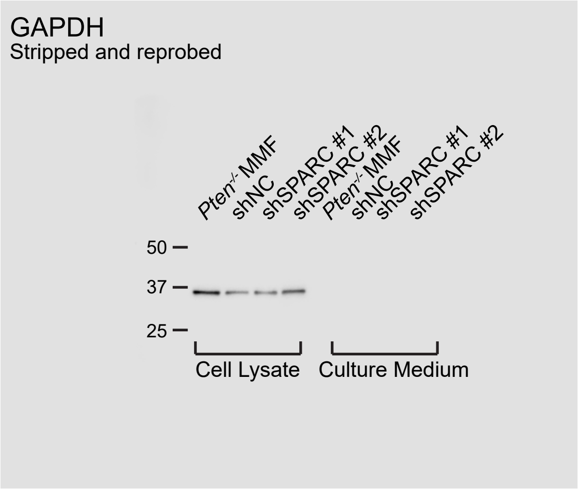

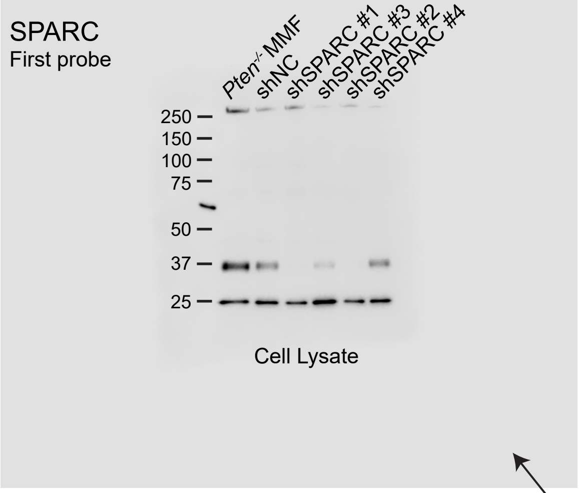

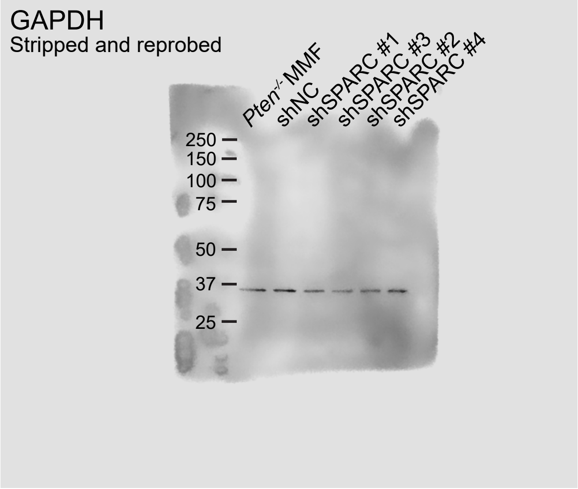

Matching set from same biological replicate

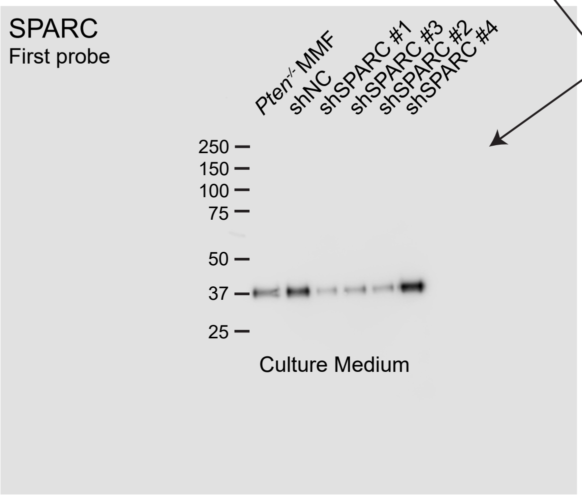

Images captured using LI-COR Odyssey FC imaging system.

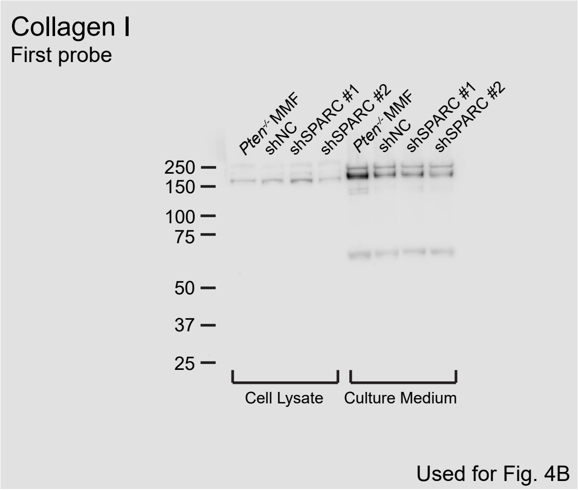

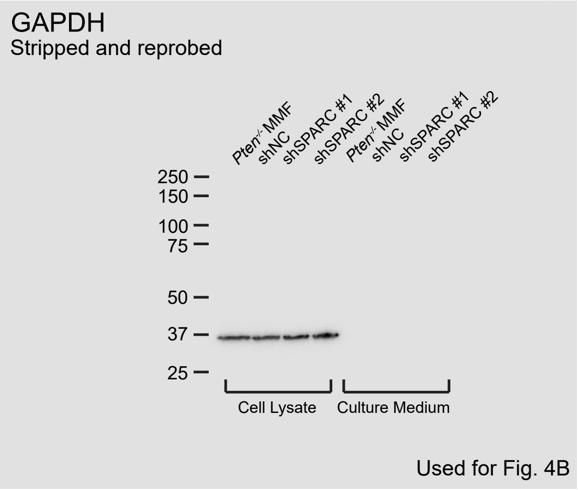

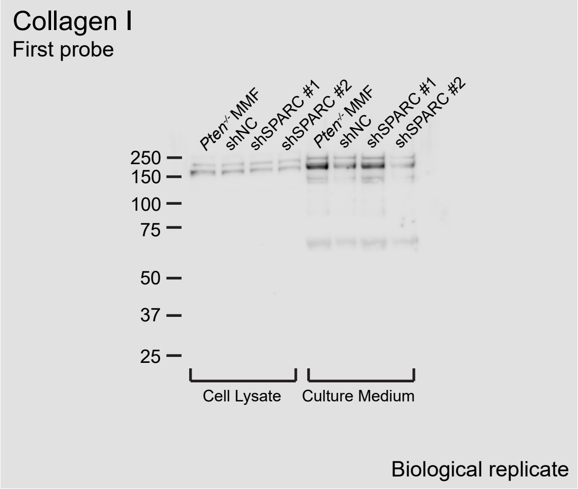

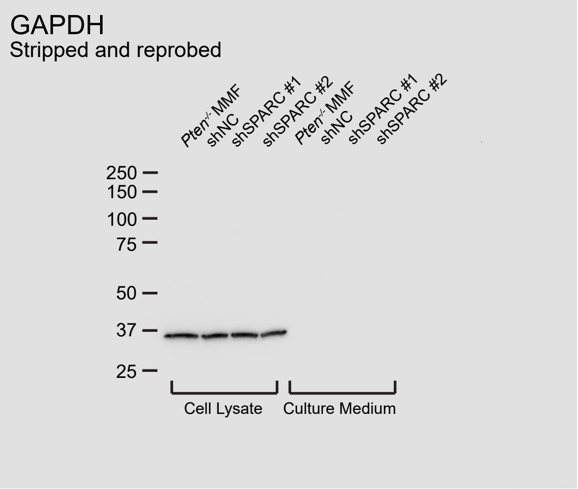

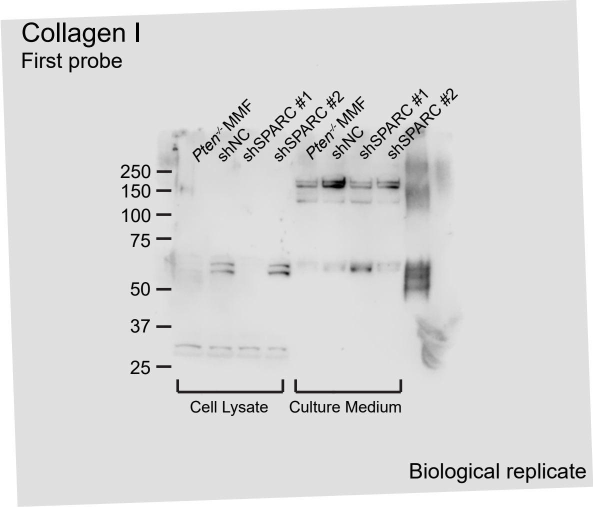

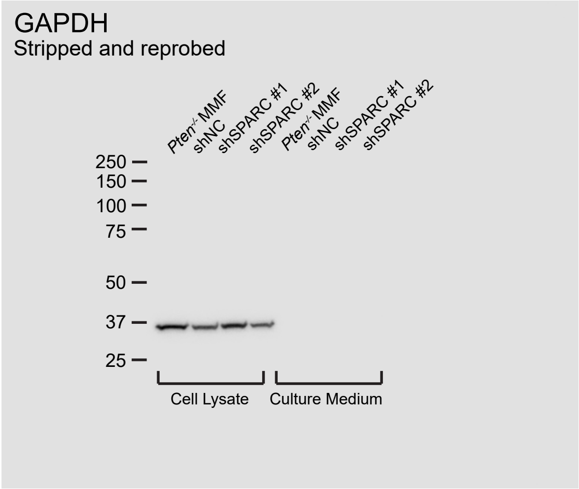

Images captured using LI-COR Odyssey FC imaging system.
