# Supplementary figures and images for: Pten regulates collagen fibrillogenesis by fibroblasts through SPARC
Source: PLoS One. 2021 Feb 3;16(2):e0245653. doi: 10.1371/journal.pone.0245653 (PMC7857610; doi:10.1371/journal.pone.0245653)

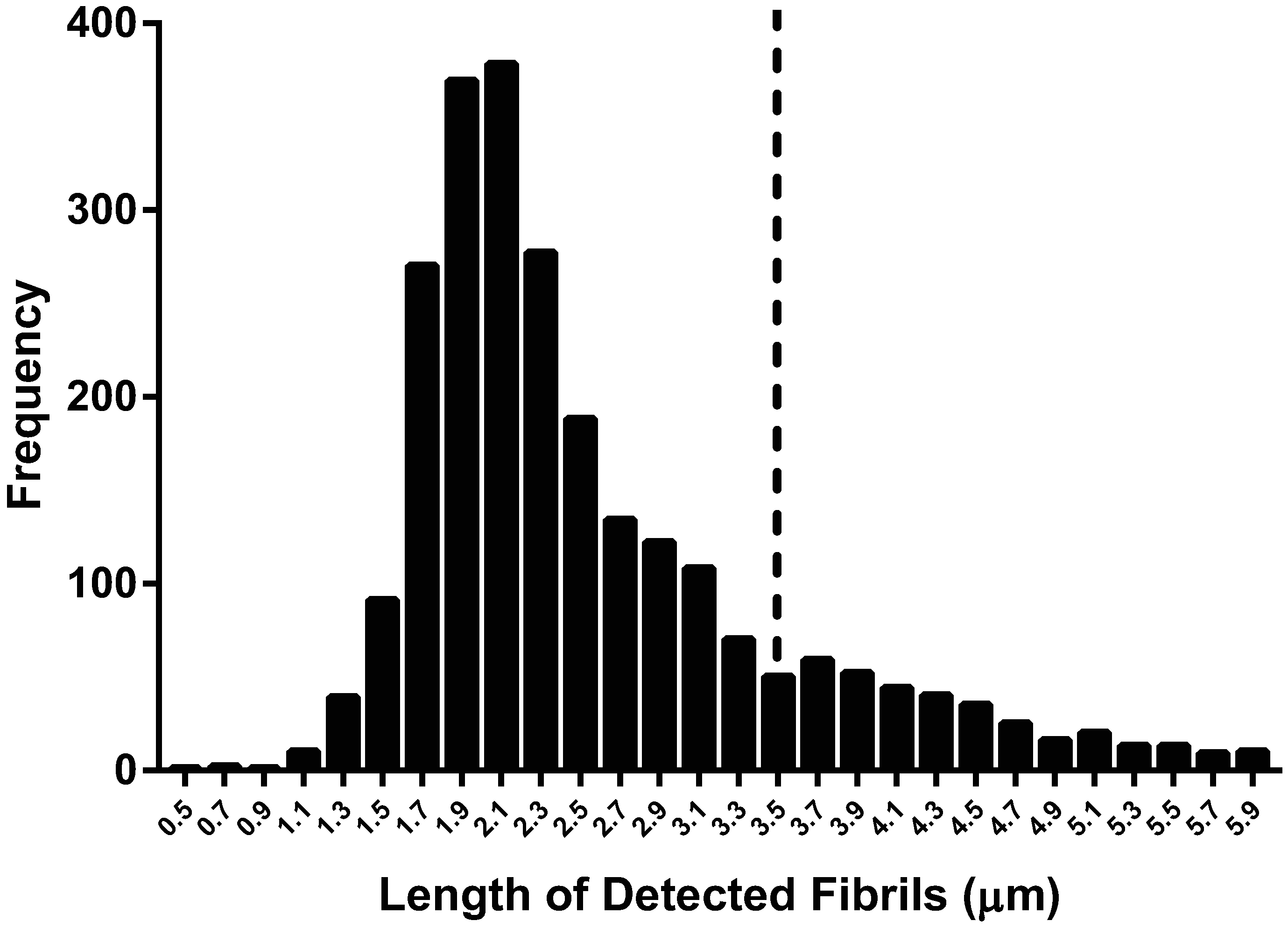

Supplement: S2 Fig — Dotted line indicates threshold used for minimum length of fibrillar adhesions. (TIF) [file pone.0245653.s002.tif]

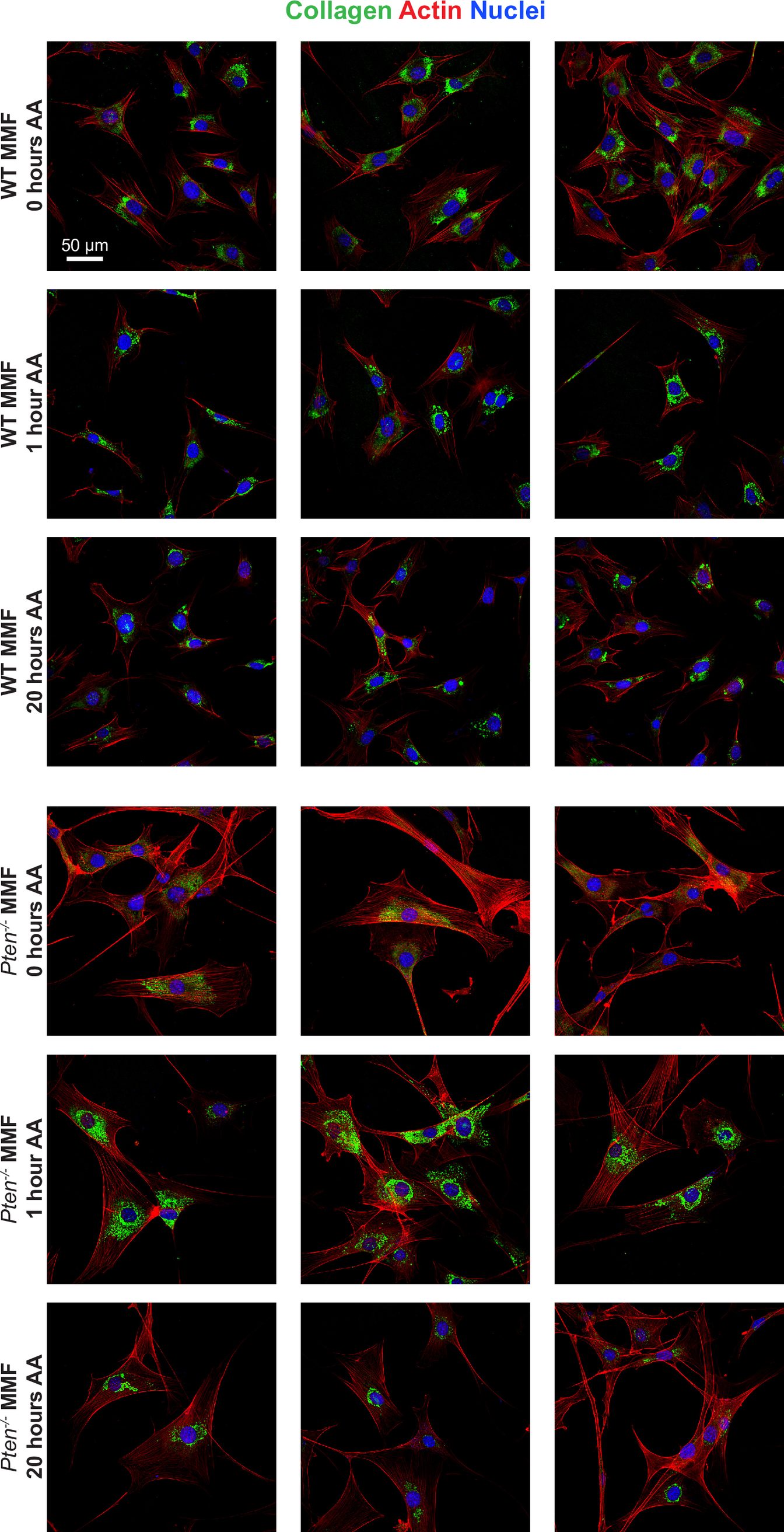

Supplement: S3 Fig — (TIF) [file pone.0245653.s003.tif]

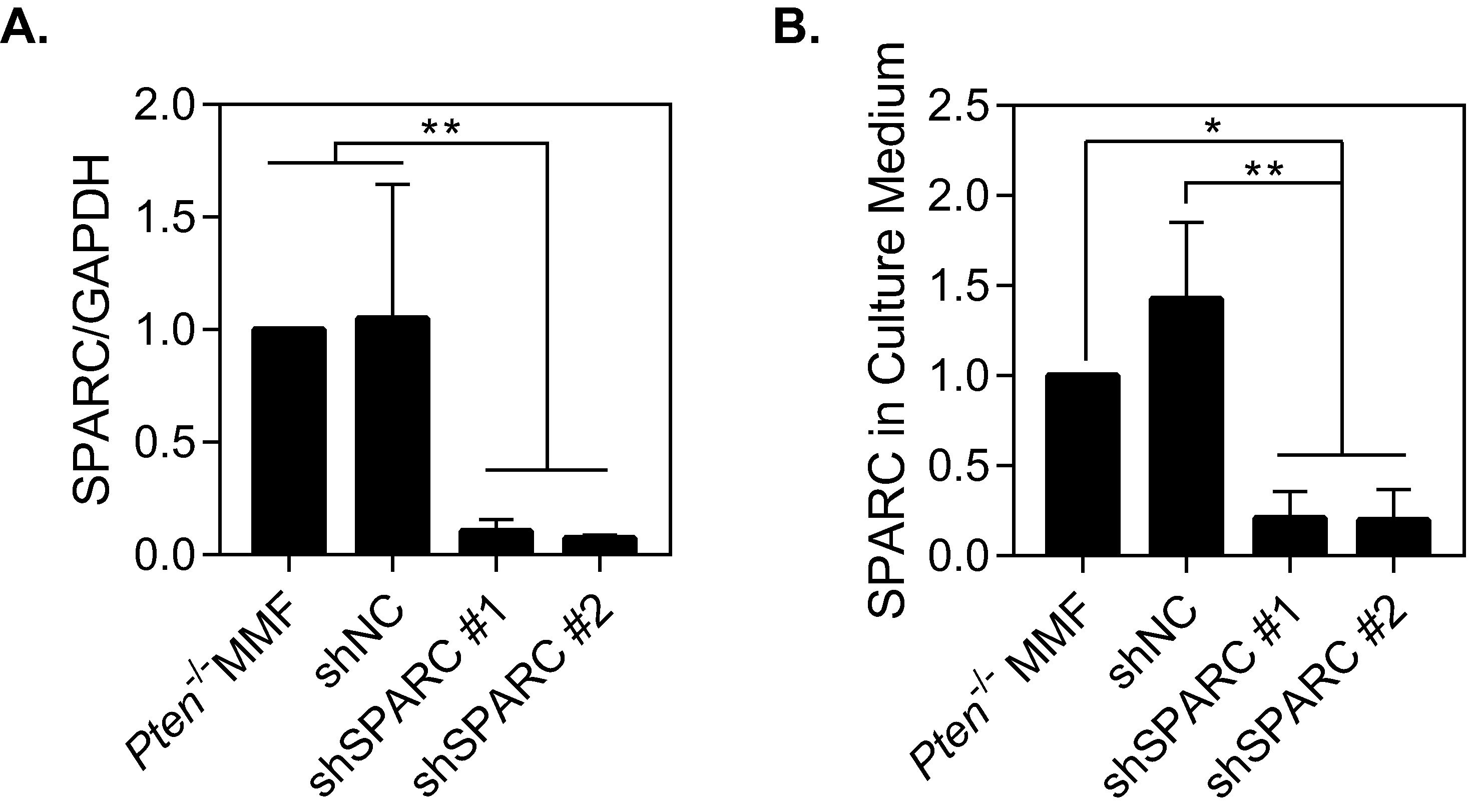

Supplement: S4 Fig — (A) Quantification of SPARC expression in the cell, normalized to GAPDH as a loading control. (B) Quantification of SPARC in the culture medium. n = 3+SD, *p<0.05, **p<0.01. (TIF) [file pone.0245653.s004.tif]

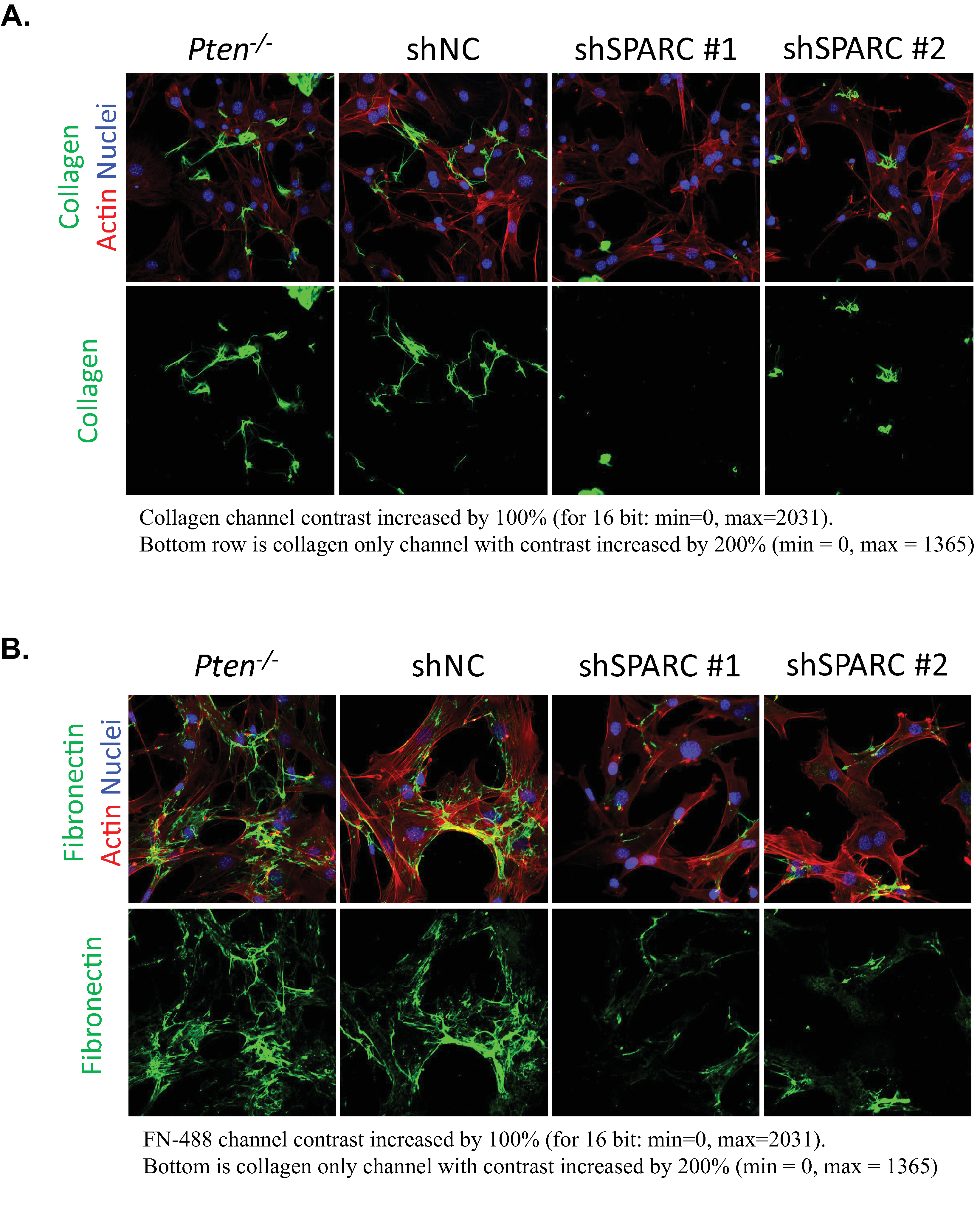

Supplement: S5 Fig — (A) Collagen fibers assembled by fibroblasts. (B) Fibronectin fibers assembled by fibroblasts. (TIF) [file pone.0245653.s005.tif]

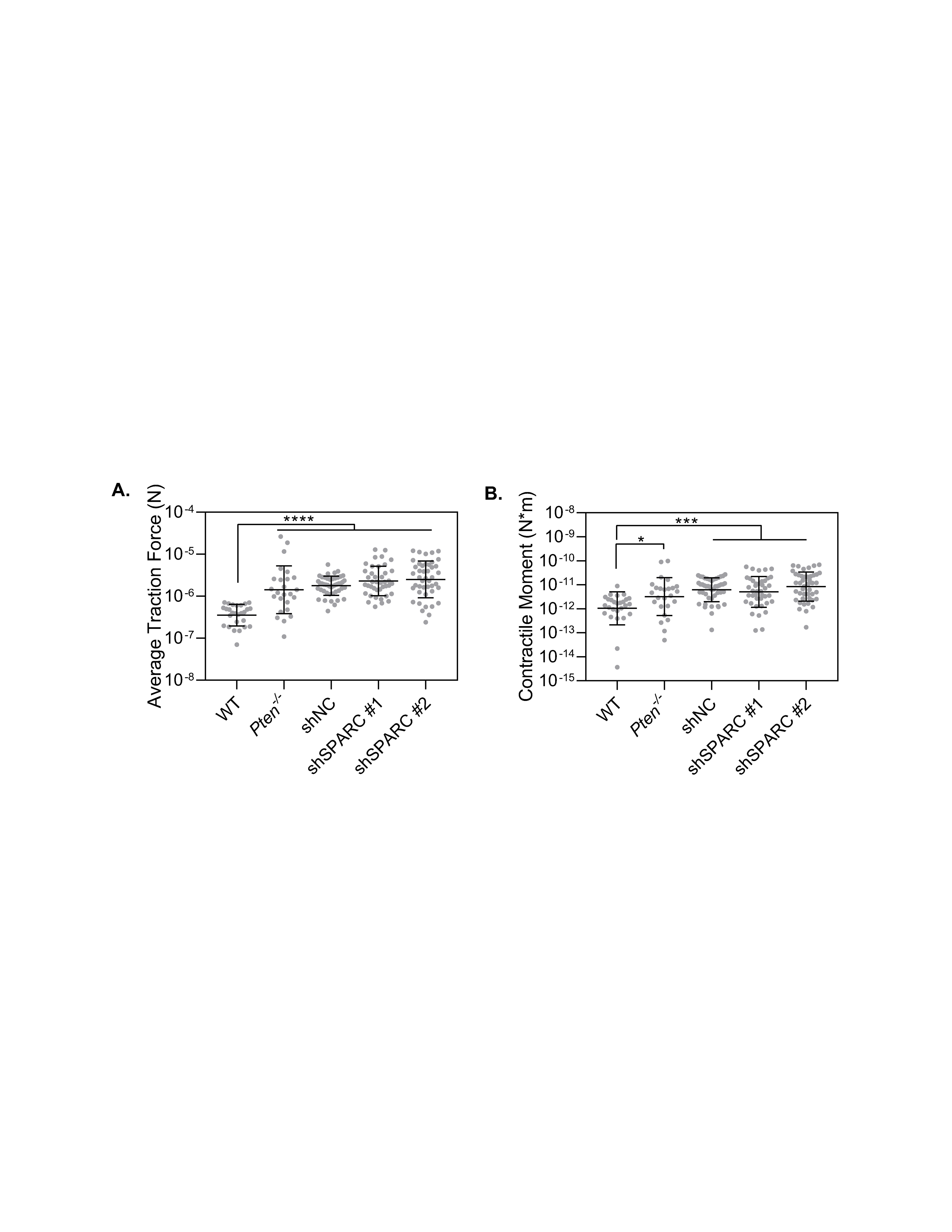

Supplement: S6 Fig — (A) Average traction force per cell, shown as mean±SD with values of individual cells plotted. (B) Net contractile moment per cell. n = 25–46 cells. *p<0.05, ***p<0.001, ****p<0.0001. (TIF) [file pone.0245653.s006.tif]
