# Supplementary material for: Pten regulates collagen fibrillogenesis by fibroblasts through SPARC
Source: PLoS One. 2021 Feb 3;16(2):e0245653. doi: 10.1371/journal.pone.0245653 (PMC7857610; doi:10.1371/journal.pone.0245653)
Supplement: Attachment — Submitted filename: Jones response to reviewers.docx [file pone.0245653.s007.docx]

We thank the reviewers for their thoughtful comments and insights. We have revised the manuscript to address their comments and provide detailed responses below.

Reviewer #1:

Major comments:

1. In Figure 2B and 4B, it is unclear why there is no band for collagen in Pten KO cell lysates, despite collagen staining in 2C and 4D. Could this be due to insufficient solubilization by RIPA lysis buffer ? Could the authors use more stringent lysis buffer to measure collagen that is deposited on to the matrix.

Author response: The differences observed in the amount of collagen may be due to the difference in timing between the immunofluorescence (1 hr of ascorbic acid treatment) and western blot (20 hr of ascorbic acid treatment) experiments. We have made the treatment times more clear in the methods and results section, as well as included new data with the immunofluorescence staining for collagen at the 0, 1, and 20 hr ascorbic acid treatment (Supplemental Figure 5), which indicates decreased collagen inside of the cells at 20 hr. This immunofluorescence staining also indicates a lack of collagen matrix deposition outside the cells at the 20 hr time point, as indicated by lack of pericellular staining. Therefore, we conclude it is unlikely that we are not solubilizing deposited matrix since little to none has been deposited at this time point. Furthermore, our lysis conditions are similar to established protocols that have been used to solubilize deposited matrix at similar time points (~24 hrs) (Soucy and Romer, Matrix Biology 2009).

2. In Figure 4B, the migration of bands for collagen are different from figure 2B. Particularly, the top band around 200 kDa, which seems to be increased in shSPARC 1 and shSPARC 2. Do these bands indicate defect in collagen deposition to the matrix in the absence of SPARC and therefore increased in the medium ? Could the authors use more stringent lysis buffer to measure collagen that is deposited on to the matrix.

Author response: The bands likely appear different due to differences in electrophoresis running times, where sometimes two distinct bands are apparent, where as with shorter run times it appears as if there is only one band. We have re-ran some of the old lysates and prepared new lysates and have been more consistent with electrophoresis times. Now, we consistently observe two bands for the upper MW bands in the collagen blots (Fig 2 and Fig 4, Supplemental Figures).

3. In figure 5A, SPARC knockdown cells seems to have more exogenous fibronectin fibrils than WT cells. Could the authors stain for endogenous cellular fibronectin or fibronectin-fibrillar adhesion markers such asintegrin α5 or integrin β1 or Tensin to see if cell-fibronectin interactions are altered in SPARC knockdown cells.

Author response: We have included an additional supplemental figure (S6), in which we have included only the fibronectin or collagen channel so that one can see this signal separate from the actin staining. We have also immunostained for the fibrillar adhesion marker tensin to investigate cell-fibronectin interactions, included in a new figure (Figure 7). We observe more fibrillar adhesions in the Pten KO fibroblasts as compared to wildtype cells, and a decrease in the adhesion number with SPARC knockdown.

Minor comments:

1. In Figure 2C, does the authors observe any significant difference in cell spreading or cell adhesion dynamics and signaling between WT and Pten KO cells ?

Author response: Yes, the PTEN KO fibroblasts spread more and the nuclei appear larger than the WT cells at the same magnification, which also agrees with a previous publication (Jones et al., Neoplasia 2019).

2. In figure 3C & 4C, what was the loading control used in the western blot quantification for culture medium samples ?

Author response: Equal amounts of concentrated medium were loaded for the western blots, and we verify similar loading and protein transfer with Ponceau S staining.

Reviewer #2.

1). The authors should state the number of primary cell isolates that contribute to each figure. For example, does the n number provided reflect separate isolates of primary cells or separate experiments with one primary isolation. The number of mice and/or mammary glands contributing to each primary isolate should be stated.

Author response: The fibroblast cells were not primary cell isolates but a spontaneously immortalized cell line previously generated in Trimboli et al., Nature 2009. We have clarified the methods to indicate that these were not primary cell isolates. N in cell culture experiments indicates the number of separate experiments that were performed, but all were with the same original cell line.

2). It is curious that collagen staining by immunohistochemistry is evident in cell layers as shown in Fig 2C and Fig.4D in PTEN -/-cells but no detection is seen in cell lysates by western blot analysis. Do the authors have an explanation for this? Was ascorbate used in the experiments from which the collagen was measured in layers and in media?

Author response: The lack of collagen bands in the original western images were likely due to the timing of the ascorbic acid treatment. Please see response to Reviewer 1, Major Comment 1. To observe collagen transport from the ER to the Golgi within the cells, cells were treated with ascorbic acid for one hour prior to fixation. For the western blots, cells were treated with ascorbic acid for 20 hr. The collagen immunostaining in supplemental figure 5 also shows lower collagen at the 20 hr treatment as compared to the 1 hr. The ascorbic acid treatment of cells lysed for western blots was mistakenly left out of the original submission, we have now corrected this in the methods and the results section.

Also, the MW for procollagen in Fig 2B is not consistent with that in Fig.4B. Are the origin of these differences known?

Author response: Please see Reviewer #1, Comment #2.

3). Overall, the authors should consider the contribution of SPARC-dependent changes in transglutaminase activity on collagen assembly in this milieu. As the collagen fiber diameter shown in Fig. 1D suggests that decreases in PTEN (and hence increases in SPARC) are associated with thicker collagen fibers. Trombetta-eSilva et al. (JBMR, 2015) and Moore Rosset et al. (JDR 2020) demonstrated that smaller collagen fibers indicative of SPARC-null tissues are reversed with inhibition of transglutaminase activity. Hence, increases in collagen fibers seen in PTEN -/-might result from increases in SPARC and modulation of transglutaminase activity. Did the authors measure levels of transglutaminase expression by mRNA? At the least, this might be added to the discussion.

Author response: We thank the reviewer for identifying this interesting potential mechanism. Because there are multiple transglutaminase family members that may contribute differently to our observations, examining transglutaminase is beyond the scope of this manuscript, however we have added this to the discussion.
